# Thyroid function is associated with insulin resistance markers in healthy adolescents with risk factors to develop diabetes

**DOI:** 10.1186/s13098-015-0011-x

**Published:** 2015-03-08

**Authors:** José de Jesús Garduño-Garcia, Eneida Camarillo Romero, Ana Loe Ochoa, Socorro Romero-Figueroa, Gerardo Huitron Bravo, Roció Torres García, Patricia Montenegro-Morales, Hugo Mendieta-Zerón

**Affiliations:** Medical Sciences Research Center (CICMED) Cuerpo académico de Salud del Universitario, 50226 Toluca, State of Mexico Mexico; High school Licenciado Adolfo López Mateos, 50226 Toluca, State of Mexico Mexico; Medical Sciences Research Center (CICMED) Cuerpo académico de biomedicina, 50226 Toluca, State of Mexico Mexico; Coordinación de Investigación en Salud, Delegación México Poniente de Instituto Mexicano del Seguro Social, 50226 Toluca, State of Mexico Mexico

**Keywords:** Thyroid, Insulin resistance, Diabetes

## Abstract

**Introduction:**

The prevalence of obesity and Type 2 diabetes mellitus (T2DM) among children and adolescents is rising. Thyroid function has been associated with insulin resistance. There is scarce information about how thyroid function could be related with cardiovascular risk or glucose homeostasis in adolescent.

**Aim:**

To analyze how thyroid function is associated with insulin resistance and another cardiovascular risk factors in healthy adolescents with risk factors to develop diabetes.

**Methods:**

A prospective cross-sectional analysis was carried out on euthyroid, adolescents. considered at high risk to develop Type 2 diabetes. Fasting blood samples were obtained. Thyroid function test and another cardiometabolic parameters were assessed. A 75 grams oral glucose tolerance test was performed to calculate insulin resistance.

**Results:**

One hundred adolescents were evaluated. The mean age was 15.9 ± 0.8 years, There is a negative correlation between Fasting insulin, post glucose load insulin and HOMA IR. There were no correlation with Matsuda index. We could not found any correlation with TSH values.

**Conclusions:**

We found a correlation between fasting insulin, HOMA IR and serum thyroid hormones, we did not find any relation with serum TSH. In euthyroid adolescents with risk factors to develop diabetes.

## Introduction

Obesity and overweight epidemic represent a big challenge all over the world. In Mexico, the National Health and Nutrition Survey (ENSANUT) 2012 data estimated a prevalence of obesity of 13.3% among adolescents (12–19 years) [1]. The chronic evolution of obesity generates devastating consequences that are associated with early mortality [2] As a consequence, the prevalence of Type 2 diabetes mellitus (T2DM) among children and adolescents is rising [3]. Since more young individuals develop T2DM, their propensity to develop diabetes related complications is higher [4]. These complications result from a continuous exposure to high plasma glucose values, and can occur even in those individuals with impaired fasting glucose (IFG) or impaired glucose tolerance [5]. Despite the relative low prevalence of T2DM at early adolescence, having high fasting plasma glucose (FPG) level, even within the normoglycemic range, is a predictor of T2DM in younger adulthood. So, it is important to find in those high risk adolescents, early biomarkers that could be associated with metabolic dysfunctions [6].

Recently, increased interest has focused on the association between thyroid dysfunction and obesity, diabetes, metabolic syndrome and other cardiovascular risk factors [7]. It is well known that patients with primary hypothyroidism have a threefold greater risk for early atherosclerosis, as shown independently for other risk factors such as atherogenic lipid profile, hypertension, and impaired endothelial function [8]. Whether subclinical hypothyroidism has an influence on the same risk factors, the association with atherosclerosis still is debated [9]. Some studies show positive results, but others do not [10]. Moreover there are authors that have postulated that this relationship could be present even in patients considered with normal thyroid function. Most of these studies have been performed in adults and much of them in geriatrics population [11]. Until now there is scarce information about how thyroid function could be related with cardiovascular risk or glucose homeostasis in adolescents [12,13]. Some of them are focused in type 1 diabetes patients [14]. Others consider patients with subclinical hypothyroidism [15,16]. There is only one study that consider euthyroid adolescents [17].

The goal of the present paper is to analyze how thyroid function is associated with insulin resistance and another cardiovascular risk factors in healthy adolescents with risk factors to develop diabetes.

## Methods

### Patients

A general meeting with first year of high school students and their parents were performed in the Lic. Adolfo Lopez Mateos UAEMex high school. First of all, a didactic explanatory talk about diabetes epidemiology and complications was given to all the participants. All patients with at least one risk factor to develop T2DM were invited to participate in the study.

We considered as T2DM risk factors; first degree family history and /or overweight or obesity, defined as a BMI ≥ 25 or 30 respectively.

### Intervention

A standardized questionnaire which included items regarding demographic information, personal and family medical history was applied to all the adolescents. Assistance by their parents to complete the questionnaire was allowed if needed. . A formal invitation were send to all the adolescents consider at high risk to develop diabetes to perform a lipid profile, thyroid function test and a 75 grams oral glucose tolerance test. A visit was programmed at the Medical Sciences Research Center (CICMED).

### Anthropometric measures

Height and weight were performed in all subjects. Anthropometric measurements were taken in a standardized manner. The blood pressure was taken using a mercury sphygmomanometer with the subjects seated for 5 min prior to measurement. A daily calibrated digital scale and stadimeter were used to measure body weight and height. The waist circumference was measured midway between the lower rib margin and the iliac crest in the horizontal plane with the patient standing.

### Laboratory

Fasting blood samples (9–12 h fast) were obtained. A 75 grams oral glucose tolerance test was performed, plasma glucose and insulin were measured before and at minute 60 and 120 after the ingestion of 75 g of glucose.

The components of the metabolic syndrome were defined in accordance with modified Cook adaptation for adolescents of the National Cholesterol, Education Program, Adult Treatment Panel III criteria (NCEP/ATPIII) [18]; abdominal obesity (waist circumference ≥ percentile 90), hypertriglyceridemia (triglycerides ≥110 mg/dl.), low HDL cholesterol (HDL cholesterol ≤40 mg/dl), elevated arterial blood pressure (blood pressure ≥ percentile 90), and abnormal fasting glucose (blood glucose ≥110 mg/dl). The metabolic syndrome was diagnosed in the presence of three or more of these factors. Insulin resistance was measured using Matsuda Index using the validated by the author 3 time points [19]. HOMA IR was calculated using the following formula: (Glucose (mg/dl) × insulin (μU/ml))/405 [20].

All blood samples were analyzed in the laboratory of the CICMED “Universidad Autonoma del Estado de México”, Toluca, Mexico. Analysis of the glucose and lipid samples was carried on the same day as blood sampling. Serum from each study subject was stored at −70 °C for later analysis.

Plasma glucose was measured with the oxidized glucose method (Randox Laboratories Ltd, Antrim, UK), triglycerides with a colorimetric method following enzymatic hydrolysis performed with the lipase technique, and HDL cholesterol (HDL-C) by the clearance method. All biomedical assays were performed with a Selectra XL instrument (Randox Laboratories Ltd, Antrim, UK). Thyroid stimulating hormone (TSH), total tri-iodothyronine (TT3), total thyroxine (TT4) free tri-iodothyronine (fT3), free thyroxine (fT4) were analyzed on a high throughput automated biochip immunoassay system, Evidence®, Randox Laboratories Ltd. Insulin was measured by Elisa through Elycsys and Cobas Analyzers Roche Diagnostics®.

### Ethics

The study was approved by the Institutional Review Board of Medical Sciences Research Center (CICMED) and informed written consent was obtained from each patient before participation.

### Statistical analyses

The descriptive analysis was performed using means and standard deviations for continuous variables. Qualitative variables were expressed as percentages. Kolmogorov-Smirnov test was performed to analyses variable distributions. Means comparison was performed using the Student’s *t*-test for quantitative variables with a normal distribution and with the Mann–Whitney *U* test for those with skewed distribution. The *χ*2 test was used to compare proportions in qualitative variables. The correlation of continuous variables was calculated using Spearman or Pearson test as appropriate. Statistical tests were performed using SPSS version 14 for Windows, Chicago, IL, USA.

## Results

A total of 525 adolescents in their first grade of high school answer the questionnaire. Oral glucose tolerance test was performed in 100 adolescents. The mean age was 15.9 ± 0.8 years, 64% of the patients were female, the mean body mass index (BMI) was 23.6 ± 5.5 kg/m^2^, and the mean waist circumference was 82.7 ± 10 cm. The prevalence of overweight and obesity (BMI > 25 kg/m^2^) was 41%. The prevalence of metabolic syndrome was 17%. The basal metabolic risk factors by sex are presented in Table 1.

**Table 1:** **Basal characteristics**

	**Male**	**Female**	***P***
**Glucose mg/dl**	92 ± 1.2	90 ± 0.7	0.08
**2 hour postload glucose mg/dl**	98 ± 4.1	102 ± 2.2	0.45
**Insulin mUI**	14 ± 1.3	13.8 ± 7.2	0.87
**HOMA IR**	2.9 ± 0.3	2.7 ± 0.1	0.58
**Matsuda Index**	14 ± 0.8	10 ± 2.2	0.72
**BMI kg/m** ^**2**^	25 ± 3.7	22 ± 5.5	0.03
**Waist cm**	86.60 ± 2.0	80 ± 1.1	0.01
**Cholesterol mg/dl**	156 ± 4.9	173 ± 4.5	0.21
**Triglycerides mg/dl**	128 ± 13	117 ± 7.7	0.42
**HDL C mg/dl**	39 ± 1.4	45 ± 1.2	0.01
**LDL C mg/dl**	101 ± 4.6	108 ± 3.9	0.22
**Systolic Blood Pressure mm Hg**	112 ± 2.6	106 ± 1.7	0.54
**Diastolic Blood pressure mm Hg**	66 ± 1.4	64 ± 0.9	0.23

A complete thyroid function test (TT4, TT3 FT3 FT4 and TSH) was measured in all subjects. There were no patients with overt thyroid disease found. Only 8 patients have TSH values > 4mUI and only 2 of them more than 5 m UI. All these patients had Thyroid hormones in normal range.

FT4 was lower in patients with HDL ≤ 40 mg/dl compared with those with HDL > 40 mg/dl 17.8 ± 0.3 vs 18.9 ± 0.3 p < 0.048 There were no difference in thyroid hormone or TSH concentration classifying by metabolic syndrome or the rest of their components or categorizing by overweight or obesity (data not shown).

When we evaluate the correlation between the metabolic variables TSH and FT4 we found that there is a negative correlation between FT4 fasting insulin, post glucose load insulin and HOMA IR. There were no correlation between FT4 and Matsuda index. We could not found any correlation with TSH values (Table 2).

**Table 2:** **Linear correlations of thyroid function parameters and metabolic markers**

		**T4**	**T3**	**FT**	**FT3**	**TSH**
Body Mass Index	R	−0.152	0.192	−0.202	0.052	−0.085
	P value	0.137	0.059	0.047	0.614	0.407
Waist	R	0.000	0.197	−0.163	−0.013	0.054
	P value	0.998	0.053	0.110	0.900	0.602
Sistolic blood Pressure	R	0.015	−0.017	−0.178	0.054	−0.172
	P value	0.884	0.871	0.087	0.610	0.098
Diastolic Blood Pressure	r	0.053	0.014	0.050	−0.025	−0.234
	P value	0.614	0.892	0.633	0.811	0.024
Total cholesterol	r	−0.020	0.140	0.042	0.198	0.119
	P value	0.845	0.166	0.681	0.050	0.239
Triglycerids	r	−0.242	0.153	−0.156	−0.006	0.084
	P value	0.016	0.133	0.124	0.950	0.409
HDL Cholesterol	r	0.108	−0.060	0.176	0.000	0.110
	P value	0.288	0.555	0.082	0.999	0.278
LDL Cholesterol	r	0.073	0.151	0.055	0.280	0.083
	P value	0.474	0.137	0.588	0.005	0.416
Fasting Glucose	r	−0.005	0.210	−0.089	0.071	−0.064
	P value	0.964	0.037	0.384	0.483	0.529
Glucose 60 min post load	r	0.006	0.105	−0.020	0.113	0.016
	P value	0.952	0.303	0.846	0.268	0.878
Glucose 120 min post load	r	−0.069	0.101	−0.073	0.141	−0.006
	P value	0.500	0.320	0.473	0.167	0.956
Fasting Insulin	r	−0.223	−0.260	−0.212	0.043	0.000
	P value	0.026	0.009	0.034	0.673	0.998
Insulin 60 minutes	r	−0.101	0.091	−0.257	−0.043	0.035
	P value	0.321	0.369	0.010	0.669	0.727
Insulin 120 minutes	r	0.032	−0.002	−0.246	−0.073	0.064
	P value	0.755	0.985	0.016	0.477	0.537
HOMA IR	r	−0.186	0.293	−0.196	0.077	−0.021
	P value	0.065	0.003	0.050	0.446	0.833
Matsuda Index	r	−0.030	−0.050	0.116	−0.034	−0.160
	P value	0.774	0.632	0.261	0.747	0.120

When we compare the effect of thyroid function test between having or not the type 2 diabetes risk factors (overweight, obesity or positive family history) we did not find any difference.

## Discussion

In the present study, we analyzed the relationship between thyroid function, insulin resistance and other cardiovascular risk factors. The study sample consisted in healthy adolescents without diabetes or known thyroid dysfunction with at least one risk factor to develop diabetes. Thyroid hormones serum concentrations seem to be similar when we stratified for the different T2DM risk factors. Moreover it exist correlation between thyroid hormones and insulin during the OGTT and HOMA IR. TSH values do not seem to correlate with insulin resistance markers.

In the last 3 decades obesity in childhood has increasing worldwide [21]. Associated with this phenomena T2DM has turn in a public health problem [22]. In adolescents the proportion between type 1 and T2DM has been modified [23]. It is estimated that T2DM in children all over the world contributing up to 45% of all cases of Diabetes. In the last years genetic background has taken an special relevance in the risk to develop T2DM [24]. It is estimated than seventy five percent of the cases have strong family history. This is of special relevant in hispanic populations. The SEARCH for Diabetes in Youth population based study found the proportion of T2DM among 10–19 years olds for Hispanics, is 33% [25]. In the present group we did not find new cases of diabetes or impaired glucose tolerance moreover the prevalence of metabolic syndrome is similar to that reported by other authors in Mexican adolescents using similar criteria [26].

Thyroid disease and diabetes mellitus are the two most common endocrine disorders in everyday practice. The possible association between both, and with other related diseases like dyslipidemia or cardiovascular disease has been postulated for several authors [27]. It is well recognized that overt thyroid dysfunction can produce dyslipidemia and insulin resistance. In the case of subclinical dysfunction is still controversial this possible association. The positive or negative results had depended on the studied populations [28]. Until the moment most of the studies has been performed in adults or geriatrics patients [29]. The correlation between thyroid hormones and insulin resistance has been tested in diabetic patients but also in subjects with normal glucose tolerance [30]. Even subtle decrease in the level of thyroid hormones within the normal range has been shown to inversely correlate with insulin resistance [31]. It is well known than aging process it is associated with the decrease of insulin sensitivity and beta cell function [32]. It exist very few information how thyroid function could be related with insulin sensitivity in early stages of the life.

Non congenital thyroid dysfunction has a relatively low prevalence in adolescents and children. A cohort study in the pediatric population, shows that initial normal or slightly elevated TSH levels are likely to remain normal or spontaneously normalize without treatment [33]. Several studies have confirmed the positive relationship between weight status and TSH levels in childhood [34,35] 10–23% of all obese children have moderately elevated TSH levels (usually between4 and 10 mIU/l), which are associated with normal free T4 (fT4) [36]. In our study we did not find correlation of the BMI with TSH or high prevalence of elevated TSH, the big difference was that we did not consider only obese patients. Sert *et al.* demonstrated that elevated TSH in the a obese group with fatty liver were positively correlate with most of the metabolic and cardiovascular risk parameters this was not shown in lean control subjects. [12].

Difference insulin resistance indexes between subclinical and euthyroid subjects were described by Maratou [37]. In our study we found correlation with thyroid hormones and HOMA IR and basal Insulin, moreover there were not with Matsuda Index. This difference could be explained in the next way. HOMA IR is a fasting insulin resistance index that is related with liver insulin resistance, on the contrary, Matsuda Index is an insulin sensitivity index that calculate in an indirect way the insulin sensitivity in muscle after a glucose load [38]. The thyroid hormones exert their physiological effects by binding to specific nuclear receptors [thyroid hormone receptors (TR)] of which the TRβ isoform is liver specific and has been considered a putative target for the treatment of dyslipidemia and fatty liver [39]. The beneficial effects of TRβ activation include lowering low-density lipoprotein (LDL) cholesterol, reducing whole body adiposity and weigh. This could hypostatized that more of the relation between insulin action and thyroid function acts at liver, in a healthy adolescents with adequate muscle metabolic function this seems to do not have big impact. This has to be confirmed with future mechanistic studies. The strength of this paper was the first in evaluate the relationship of thyroid function and insulin resistance with a dynamic test in euthyroid adolescents with diabetes risk factors, the weakness is the cross sectional design.

In summary we found a correlation between fasting insulin, HOMA IR and serum thyroid hormones, we did not find any relation with serum TSH. In euthyroid adolescents with risk factors to develop diabetes. We considered that most research has to be done in children and adolescents to find early markers than can predispose to develop T2DM which will be come in the future the most serious health problem all over the world.
